# A Polymeric Protein Induces Specific Cytotoxicity in a TLR4 Dependent Manner in the Absence of Adjuvants

**DOI:** 10.1371/journal.pone.0045705

**Published:** 2012-09-24

**Authors:** Paula M. Berguer, Vanina A. Alzogaray, Andrés Hugo Rossi, Juliana Mundiñano, Isabel Piazzon, Fernando A. Goldbaum

**Affiliations:** 1 Fundación Instituto Leloir, IIBBA, Consejo Nacional de Investigaciones Científicas y Técnicas (CONICET), Buenos Aires, Argentina; 2 IMEX-CONICET, Laboratorio de Medicina Experimental, Academia Nacional de Medicina, Buenos Aires, Argentina; National Council of Sciences (CONICET), Argentina

## Abstract

Lumazine synthase from *Brucella* spp. (BLS) is a highly immunogenic decameric protein. It is possible to insert foreign peptides or proteins at its ten-amino acid termini. These chimeras elicit systemic and oral immunity without adjuvants, which are commonly needed in the formulation of subunit-based vaccines. Here, we show that BLS induces the cross presentation of a covalently attached peptide OVA_257–264_ and a specific cytotoxic response to this peptide in the absence of adjuvants. Unlike other subunit-based vaccines, this chimera induces rapid activation of CTLs and a specific cytotoxic response, making this polymeric protein an ideal antigen carrier for vaccine development. Adoptive transfer of transgenic OT-I T cells revealed efficient cross presentation of BLS-OVA_257–264_ *in vivo*. BLS-OVA_257–264_ immunization induced the proliferation of OVA_257–264_-specific CD8+ lymphocytes and also increased the percentage of OVA_257–264_-specific CD8+ cells expressing the early activation marker CD69; after 5 days, the percentage of OVA_257–264_-specific CD8+ cells expressing high levels of CD44 increased. This cell subpopulation showed decreased expression of IL-7Rα, indicating that BLS-OVA_257–264_ induced the generation of CD8+ effector cells. BLS-OVA_257–264_ was cross presented *in vitro* independently of the presence of a functional TLR4 in the DCs. Finally, we show that immunization of wild type mice with the chimera BLS-OVA_257–264_ without adjuvants induced a strong OVA_257–264_-specific effector cytotoxic response. This cytotoxicity is dependent on TLR4 as is not induced in mice lacking a functional receptor. These data show that TLR4 signaling is necesary for the induction of a cytotoxic response but not for antigen cross presentation.

## Introduction

The processing and presentation of a protein by antigen presenting cells plays a decisive role in the specific recruitment and activation of distinct T cell subsets during an immune response. Professional APCs, such as dendritic cells (DCs), have the capacity to take up exogenous antigens and shunt them into the class I pathway for presentation to CD8+ cells in a process termed cross presentation [1]–[3]. Through this process, the activation of CD8+ lymphocytes, which is essential for the elimination of many intracellular pathogens including some viruses and many bacteria, is accomplished. Few isolated exogenous antigens have some intrinsic characteristics that give them the ability to stimulate a response upon their presentation on MHC I. These characteristics include antigen association with cells, association with lipids, epitope repetitiveness, fusogenic potential and antigen stability [4]–[7] and in general they are particulate antigens [8]. A large body of evidence indicates that the half life of an antigen is a critical parameter that influences its likelihood of being displayed on the MHC I through cross presentation [4], [9]–[12]. Certain TLR ligands activate DCs, inducing their cross presentation and a cytotoxic response [13], [14]. For soluble protein antigens, it has previously been shown that cross priming is more efficient if an adjuvant such as a CpG oligonucleotide is chemically linked to the antigen [15]–[18]. Even for virus-like particles, more potent CTL responses are obtained when nonmethylated CpG motifs are packaged into the particles rather than coinjected [19]. However, not all TLR ligands are capable of inducing antigen cross presentation, and in some cases, TLR signaling would inhibit antigen uptake for presentation on the MHC I [20].

The enzyme lumazine synthase from *Brucella* spp. (BLS) is a highly immunogenic protein [21]–[23]. The efficacy of several BLS vaccines has been shown [24]–[26]; it acts as a potent oral or systemic immunogen when injected as a protein or as a DNA vaccine [24], [27]. BLS has been used as a protein carrier of foreign peptides and proteins [28], [29]. Crystallographic and spectroscopic solution studies have allowed us to determine the dissociation and unfolding mechanisms of the decameric structure, serving as a platform for protein engineering purposes [30], [31]. BLS folds into a highly stable dimer of pentamers and displays an intertwined structure, where approximately 45% of the surface of each monomer is engaged in monomer-monomer and pentamer-pentamer contacts [30], [31]. Of note, BLS is resistant to urea denaturation and is more stable to thermal denaturation than the cholera toxin. BLS activates DCs *in vitro*, increasing the levels of co-stimulatory molecules and the secretion of proinflammatory cytokines, and recruits DCs *in vivo*, both effects in a TLR4-dependent manner [32]. Although soluble proteins are poor substrates for cross presentation, we decided to study the ability of BLS to induce antigen cross presentation because it activates DCs through TLR4 [32], it forms a protein particle of middle size [31] and is also remarkably stable and is resistant to protease hydrolysis (unpublished observations). In this work, we show that immunization with the chimera BLS-OVA_257–264_ induces the cross presentation of peptide OVA_257–264_, generating rapid proliferation and activation of specific CD8+ lymphocytes. An *in vivo* assay demonstrated that this immunogen generates in normal mice a remarkable, specific cytotoxic response that eliminates a significant percentage of OVA_257–264_-loaded cells. Our results also show that in the absence of TLR4 the cross presentation is induced but the cytotoxic response is abolished.

## Materials and Methods

### Mice

C57BL/6J mice and congenic OT-I mice that possess a transgenic TCR specific for H-2K^b^ and OVA_257–264_ (SIINFEKL sequence) [33], C57BL/10J (wild type) and C57BL/10ScNJ mice (carrying a spontaneous deletion of the Tlr4 gene) were obtained from The Jackson Laboratory and were bred in the animal facility of the Experimental Medicine Laboratory, IMEX-CONICET, Academia Nacional de Medicina. All mice were bred under specific pathogen-free conditions and were used at 8–10 wk of age.

### Ethics Statement

Mice were housed and treated according to the policies of the Academia Nacional de Medicina and the National Institutes of Health Guide for the Care and Use of Laboratory Animals [34]. All efforts were made to minimize suffering and the procedures were approved by the Ethics Committee of the Academia Nacional de Medicina.

### Generation And Purification Of Proteins

#### BLS

Cloning, recombinant expression, and purification of BLS protein were performed as described previously [28], [35]. Briefly, the BLS gene was cloned into the pET11a vector (Novagen) and transformed and expressed as inclusion bodies in the BL21 (DE3) strain of *Escherichia coli*. The inclusion bodies were solubilized in 50 mM Tris, 5 mM EDTA, and 8 M urea (pH 8.0) overnight at room temperature with agitation. The solubilized material was refolded by dialysis against PBS containing 1 mM DTT for 72 h. This preparation was purified with a Q-Sepharose column in a fast performance liquid chromatography apparatus (Amersham Biosciences) using a linear gradient of NaCl between 0 and 1 M in 50 mM Tris (pH 8.5). The peak enriched with BLS was further purified on a Superdex-200 column with PBS and 1 mM DTT. The purity of the BLS preparation was determined using 15% (w/v) SDS-PAGE. BLS was concentrated (to 2 mg/ml), frozen in liquid N_2_, and stored at −20°C. Purified BLS was detoxified by incubation with 1 mg of BLS with 500 µl of polymyxin B-agarose (PMB-agarose) overnight twice at 4°C, as previously described [32]. LAL test was performed in order to assure that BLS and BLS-OVA_257–264_ preparations were free of LPS.

#### BLS-OVA_257–264_

The procedure used to generate BLS chimeras was previously described [28]. To generate BLS-OVA_257–264_, the coding sequence for chicken OVA peptide 257–264 was inserted at the N terminus of BLS in vector pet11a. The vector was transformed into and expressed in BL21 (DE3) *E. coli*. The chimera was purified from bacterial cytoplasm. The purification steps were the same as those for BLS. The purity of the samples was determined by SDS-PAGE. BLS-OVA_257–264_ was detoxified with PMB-agarose as described for BLS.

### Naive T Cell Purification

Inguinal, axillary, popliteal and mesenteric lymph nodes were harvested from OT-I mice. They were then pooled, disrupted and passed through a 30-µm pore filter (Pre-separation filtres, Miltenyi Biotec) to obtain a single-cell suspension. Purified CD8+ T cells were obtained by negative selection using MACS (magnetic cell sorting, Miltenyi Biotec). Briefly, cells were coated with biotin-labeled antibodies specific for CD4, CD11b, CD45R and Ter119. Anti-biotin magnetic MicroBeads (Biotin-Antibody cocktail, Miltenyi Biotec) were added to the cells, which were then passed over LS separation columns attached to the MACS magnet. All steps in the process were performed under sterile conditions. The cells that did not bind to the column were collected and assessed by FACS analysis to be 98% CD8+.

### Fluorescent Labeling of OT-I CD8+ Cells

CFSE labeling was performed as previously described [36]. Briefly, purified CD8+ T cells from OT-I mice were resuspended in PBS containing 0.3% BSA (Sigma, St. Louis, MO) to a concentration of 10^7^ cells/ml. For fluorescence labeling, 10 µM of a CFSE (Molecular Probes, Eugene, OR) stock solution was incubated with 10^7^ cells for 15 min at 37°C. The cells were incubated twice with 10% FBS in RPMI. FACS analysis was performed to ensure that all of the cells were labeled.

### Adoptive Transfer and Immunizations

A total of either 5 or 10×10^6^ purified and CFSE-labeled naive OT-I CD8+ cells in 0.3 ml PBS were transferred via tail vein injection into age- and sex-matched, 6- to 8-week old naive C57BL/6J recipients. Recipient mice were rested for 1 h before subcutaneous immunization in the tail base with 20 µg of BLS-OVA_257–264_ in PBS, 46 µg of OVA in IFA or in PBS, 1 µg of OVA_257–264_ in IFA or in PBS, or 19 µg of BLS in PBS. The doses of BLS, OVA and OVA_257–264_ were calculated to administer the same mass of BLS and OVA_257–264_ as that in the immunization with BLS-OVA_257–264_. Either 20 hours or 5 days later, draining lymph nodes (inguinal and paraaortic, separately) were removed and processed for FACS analysis.

### *In Vitro* Cross Presentation Assay

Splenic DCs were obtained from C57BL/6J, C57BL/10J or C57BL/10ScNJ mice by positive selection of CD11c+ cells using magnetic sorting (MACS System, Miltenyi Biotec). Cell purity was >95%, as assessed by CD11c staining. Purified DCs were plated at 4×10^5^ cells in 0.2 ml/well of a 96-well plate in RPMI 1640 medium and incubated at 37°C for 18 h with 200 µg of BLS-OVA_257–264_, 50 µg of OVA or with PBS. The phenotype of the DCs was determined by staining with PE-conjugated anti-CD11c (HL3), FITC-conjugated anti-CD40 (HM40-3), FITC-conjugated anti-CD80 (16–10A1) mAbs (BD Pharmingen) and subjected to FACS analysis. DCs were fixed with 0.5% paraformaldehyde in PBS for 15 min at room temperature and then washed extensively with complete medium. A total of 2×10^5^ CD8+ cells from lymph nodes of OT-I mice (previously stained with CFSE) were then added to each well and incubated for 20 h at 37°C. The CD8+ T cell response was assessed by determining the level of CD69 expression and CFSE staining by flow cytometry.

### Flow Cytometry Analysis of OT-I Cells

Mice were sacrificed at the indicated times after adoptive transfer and immunization. Inguinal and paraaortic lymph node cells were harvested separately, counted by trypan blue dye exclusion to determine total viable cell counts and stained with anti-CD8 antibodies. Transferred OT-I cells were detected as CD8+ CFSE+ cells. Cells were stained with the following monoclonal antibodies (BD Pharmingen): PE-conjugated anti-CD69 (cat# 553237, clone H1.2F3), PE-conjugated anti-CD127 (cat# 552543, clone SB/199), PE- and Cy-chrome 5 (Cy)-conjugated anti-CD8 (cat# 553033 and 553034, respectively, clone 53-6.7), Cy-conjugated CD44 (cat# 553135, clone IM7) or the appropriate isotype controls and subjected to FACS analysis. OT-I cells from the *in vitro* assay (detected as CD8+ CFSE+ cells) were stained with PE-conjugated anti-CD69 and Cy-conjugated anti-CD8 monoclonal antibodies. Cells were acquired on a FACScalibur cytometer (BD Biosciences, Ref 342976, Mfd Aug 2006). Data were analyzed using CellQuest software (BD Immunocytometry Systems).

### CTL Assay

C57BL/6J mice were immunized s.c. with BLS-OVA_257–264_ or controls and after 6 days they were i.v. transferred with target cells. Eighteen hours after the inoculation of target cells, draining lymph nodes were removed and processed for FACS analysis. These three steps are described below.

#### Immunization

C57BL/6J mice were immunized s.c. in the base of the tail with 50 µg of BLS-OVA_257–264_ in 100 µl of sterile PBS or with AlOH, CFA or IFA. Other groups of mice were immunized with 47.5 µg of BLS in PBS, with 2.5 µg of OVA_257–264_ in PBS or 115 µg of OVA in PBS or AlOH. The doses of BLS, OVA and OVA_257–264_ were calculated to administer the same mass of BLS or OVA_257–264_ as that in the immunization with BLS-OVA_257–264_.

#### Inoculation of target cell

The *in vivo* CTL assay was performed as reported previously [37], [38]. The spleen and lymph nodes (popliteal and inguinal) from naive C57BL/6J mice were removed for use as target cells. Mononuclear cells from spleen cells were isolated using a Ficoll-Hypaque gradient. These cells were mixed with the lymph node cells and then were equally divided into two populations. One was pulsed with 20 µg/ml of purified OVA_257–264_ peptide (NeoMPS, Strasbourg, France) for 30 min at 37°C and then labeled with a high concentration of CFSE (10 µM). The other population was not pulsed and was labeled with a low concentration of CFSE (0.7 µM). Equal numbers of cells from each population (2×10^7^) were mixed together and adoptively transferred i.v. into naive and immunized C57BL/6J mice at 6 days post-immunization.

#### Flow cytometry analysis and calculation of specific lysis percentage

Inguinal and paraaortic lymph nodes were removed and processed for FACS analysis 18 h after inoculation with CFSE-labeled cells. Each population was distinguished by its respective fluorescence intensity. The percentage of specific cytotoxicity was calculated using the following formula: % specific lysis = [1−(r control/r immunized)]×100 and r is calculated as r = % CFSE^low^/% CFSE^high^, where CFSE^high^ represents the number of peptide-pulsed cells and CFSE^low^ represents the number of unpulsed cells recovered from either control or immunized mice [38].

To test the role of TLR4 in the cytotoxic response, the CTL assay was performed with C57BL/10ScNJ or C57BL/10J mice as recipients for the transfer of OVA_257–264_-loaded splenocytes from C57BL/10J mice.

### Determination of IFN-γ

#### RT-PCR

C57BL/10J or C57BL/10ScNJ were given a 50 µg of BLS s.c. injection in the right hind footpad. At 48 h total RNA from poplytheal lymph nodes was obtained with the RNeasy Mini Kit (Qiagen Inc., Valencia, Calif.) following the manufacturer's instructions. The expression of IFN-γ was determined by RT-PCR using specific primers, the Avian Myeloblastosis Virus Reverse Transcriptase and OligodTs (Invitrogen Life Technologies) following the manufacturer's instructions. The reaction was performed using equal ammounts of cDNA and the products were analyzed by BrEt stained 2% agarose gels. The quantification of the bands was performed with Scion Image NIH programme and the relative expression of IFN-γ was determined in comparison with the level of actin. Data are expressed as the fold increase of mRNA in draining versus non-draining lymph nodes.

#### ELISA

C57BL/10J or C57BL/10ScNJ mice were i.p. inoculated with 50 µg of BLS 3 times with intervals of 4 days. Splenocytes were cultured and exposed to 50 µg of BLS. IFN-γ content in the supernatants after 24 h of stimulation was determined using ELISA (OptEIA set; BD Pharmingen), following the manufacturer's instructions. The reaction was developed by adding 50 µl of a solution containing 2 µg/µl ortho-phenylenediamine and 0.03% H_2_O_2_ in 0.1 M citrate-phosphate buffer and was stopped with 50 µl of 4 N H_2_SO_4_. The final color was read at 492 nm in an ELISA reader (SLT Labinstruments). The detection limit was 31.3 pg/ml.

### Statistical Analysis

Results are expressed as means + SD. Levels of significance were determined using two-tailed Student's *t*-test, and a confidence level of greater than 95% (p<0.05) was used to establish statistical significance.

## Results

### BLS-OVA_257–264_ Induces the Specific Proliferation of OT-I CD8+ T Cells

**Figure 1:**
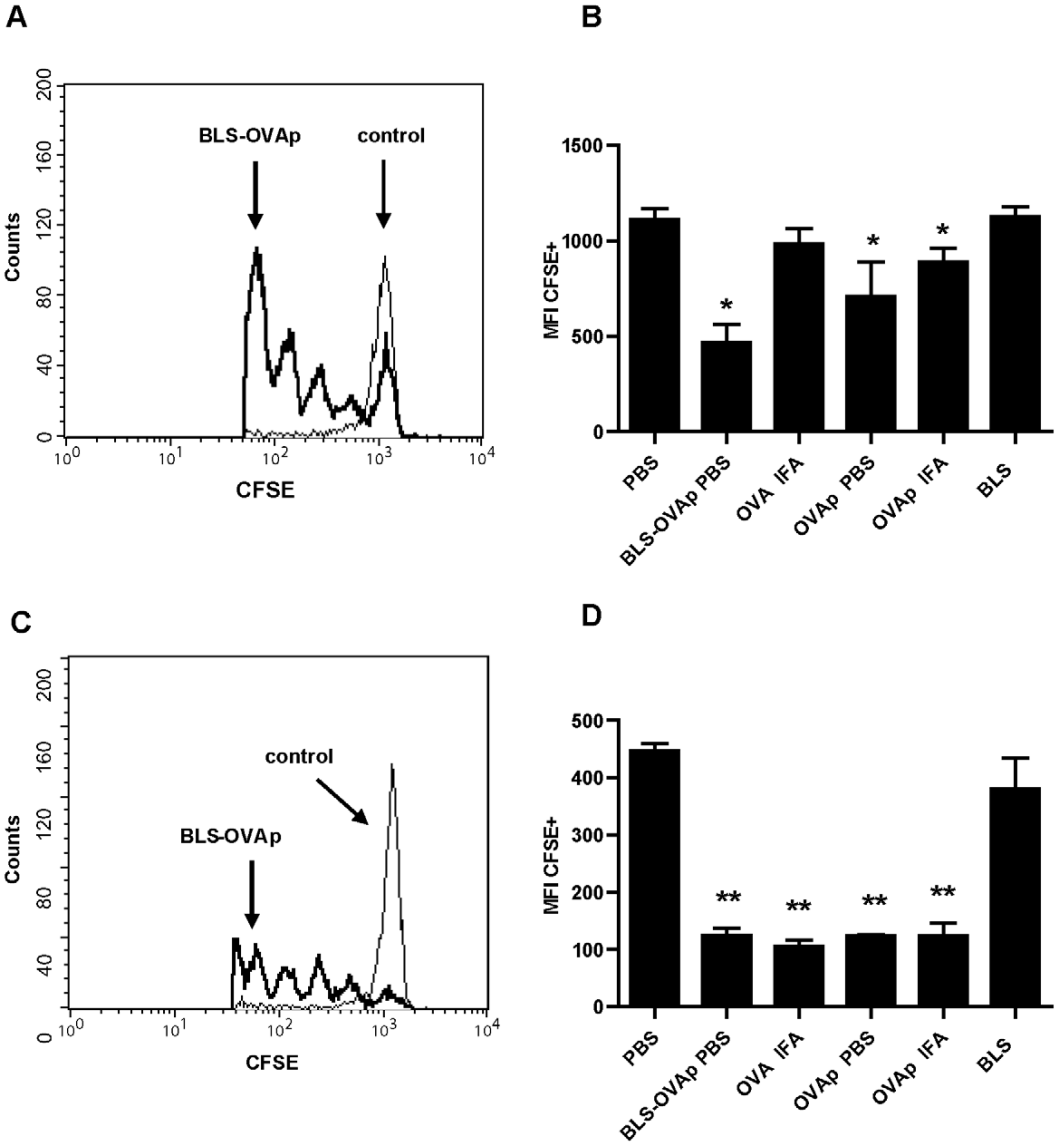
Specific CD8+ proliferation induced by BLS-OVA_257–264_. C57BL/6J mice received CFSE-labeled OT-I CD8+ cells and were then immunized s.c. with BLS-OVA_257–264_ (BLS-OVAp), OVA in IFA, OVA_257–264_ (OVAp), OVAp in IFA, BLS or PBS (control). After either 20 h or 5 days, draining lymph nodes were removed, and the CFSE label was analyzed by FACS. The intensity of CFSE fluorescence in CD8+ cells (gated on CFSE+ cells) as an indicator of cell proliferation is shown. Histograms depict representative results for BLS-OVAp and control at 20 h (A) or 5 days (C). Bars represent CFSE mean fluorescence intensity (MFI) + SD for all groups (n = 10) at 20 h (B) or 5 days (D). **p<0.0001 or *p<0.01 compared to control. Data of three independent experiments have been pooled.

In order to analyze the ability of BLS to induce the cross presentation of covalently attached peptides, we generated the chimera BLS-OVA_257–264_, in which BLS displays 10 copies of the peptide OVA_257–264_. CD8+ cells from OT-I transgenic mice that recognize OVA_257–264_ in the context of MHC I were stained with CFSE and inoculated intravenously in C57BL/6J congenic mice (adoptive transfer). These mice were then immunized with the chimera BLS-OVA_257–264_. After either 20 h or 5 days, the draining lymph nodes were removed, and FACS analysis was performed. BLS-OVA_257–264_ immunization induced the proliferation of specific CD8+ lymphocytes after 20 h (Fig. 1 A and B). Immunization with OVA in IFA did not induce the proliferation of specific CD8+ cells after 20 h. Proliferation of OT-I CD8+ cells from mice immunized with BLS-OVA_257–264_ without adjuvant continued after 5 days (Figure 1 C and D). At this time, the proliferation of specific CD8+ cells was also observed in mice immunized with OVA in IFA. Immunization with OVA_257–264_ in PBS or in IFA induced the proliferation of OT-I CD8+ cells in both analyzed times. CD8+ cells from mice immunized with BLS alone did not proliferate, indicating the specificity of the response.

### BLS-OVA_257–264_ Activates CD8+ T Lymphocytes

**Figure 2:**
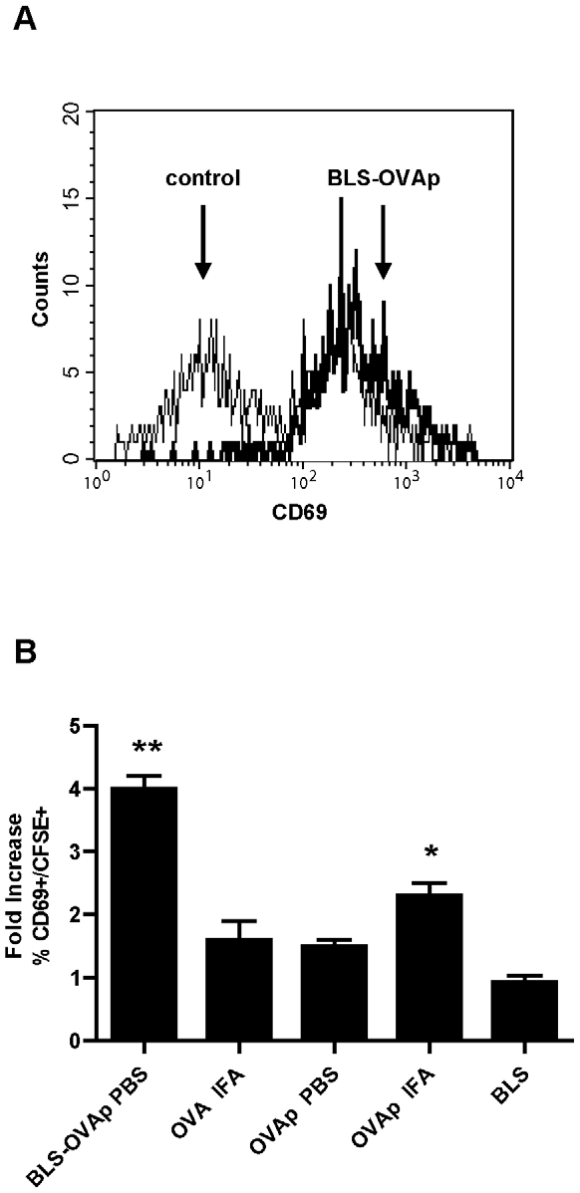
Early activation of specific cells induced by BLS-OVA_257–264._ C57BL/6J mice received CFSE-labeled OT-I CD8+ cells and were then immunized s.c. with BLS-OVA_257–264_ (BLS-OVAp), OVA in IFA, OVAp, OVAp in IFA, BLS or PBS (control). At 20 h, draining (inguinal) lymph nodes were removed, and CD69 fluorescence was analyzed by FACS. A: Histograms show CD69 expression in CFSE+ cells. B: Percentage of CFSE+ cells expressing CD69. Bars represent the fold increase of the mean % + SD (n = 9). **p<0.001 and *p<0.05 compared to control. Data of two independent experiments have been pooled.

The phenotype of OT-I cells was analyzed in order to study their activation state. At 20 h of BLS-OVA_257–264_ immunization, the expression of the early activation antigen CD69 was examined in CFSE-labeled cells from lymph nodes of recipient mice. The percentage of OT-I CD8+ cells that expressed CD69 (%CD69+/CD8+) was significantly increased. Figure 2A shows a representative histogram of the expression of CD69 in CFSE+ (OT-I CD8+) cells. Figure 2B shows the fold increase of the %CD69+ in CFSE+ cells. An increment in the percentage of CD69+/CD8+ cells in mice immunized with OVA_257–264_ in IFA was also observed, although it was significantly lower than that in mice immunized with the chimera. Immunization with OVA in IFA, OVA_257–264_ in PBS or with BLS did not alter the percentage of CD69+/CD8+ cells.

**Figure 3:**
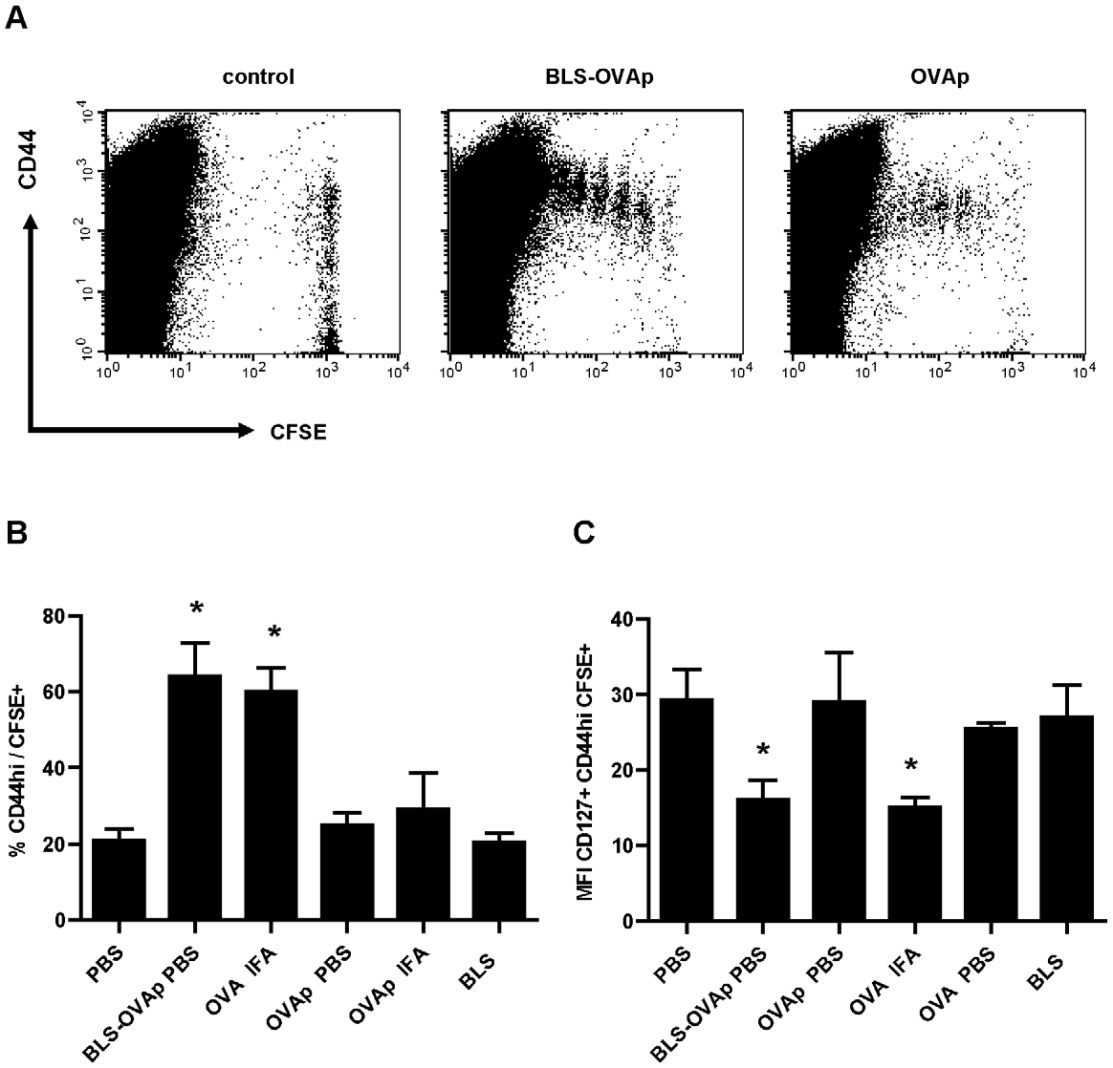
Changes induced by BLS-OVA_257–264_ in the phenotype of CD8+-specific cells. C57BL/6J mice received CFSE-labeled OT-I CD8+ cells and were then immunized s.c. with BLS-OVAp, OVA in IFA, OVAp, OVAp in IFA, BLS or PBS (control). After 5 days, inguinal lymph nodes were removed, and the expression of CD44 and CD127 was analyzed by FACS. A: Dot plots show CD44 and CFSE expression in lymph node cells. B: Bars represent the percentage+SD of CFSE+ cells expressing high levels of CD44+ (n = 10); *p<0.001 compared to control. C: Bars represent the MFI+SD of CD127 in CD44^high^ CFSE+ cells (n = 10); *p<0.05 compared to control. Pooled data of three experiments are shown.

The generation of activated/memory CD8+ T cells was assessed on day 5 after adoptive transfer and immunization by examining the expression of CD44 (Fig. 3A and B). BLS-OVA_257–264_ induced a significant increase in the percentage of OT-I CD8+ cells expressing high levels of CD44. This also occurred in mice immunized with OVA in IFA. Immunization with OVA_257–264_ in PBS, OVA_257–264_ in IFA and BLS in PBS did not increase the levels of CD44. In contrast to naive T cells, lymphocytes that encounter the antigen or memory T cells exhibit differential expression of certain surface markers, including CD69, CD44, CD62L and CD127 [16], [39]–[42]. Cytokine IL-7 is essential for the survival of CD8+ T cells [43]. The alpha chain of its receptor, CD127, is constitutively expressed on the surface of CD8+ cells and is downregulated in recently activated effector cells [40]. To study whether BLS-OVA_257–264_ immunization induces the differentiation of CD8+ lymphocytes toward a phenotype of memory cells with effector functions, we analyzed the expression of CD127 in transferred cells. Immunization with BLS-OVA_257–264_ induced the downregulation of CD127 in cells expressing high levels of CD44 (Fig. 3C). This effect was also observed in mice immunized with OVA in IFA. Immunization with either OVA_257–264_, OVA or BLS did not induce this downregulation. These results show that BLS-OVA_257–264_ stimulation stimulates the generation of CD8+ effector cells in the absence of adjuvants.

Taken together, these results reveal that the association of peptides at the structure of BLS induces their cross presentation, generating the activation and proliferation of specific CD8+ cells. When stimulated with the chimera, these lymphocytes have the phenotype of effector cells.

### Efficient Cross-Presentation of BLS-OVA_257–264_ in TLR4-Defficient DCs

**Figure 4:**
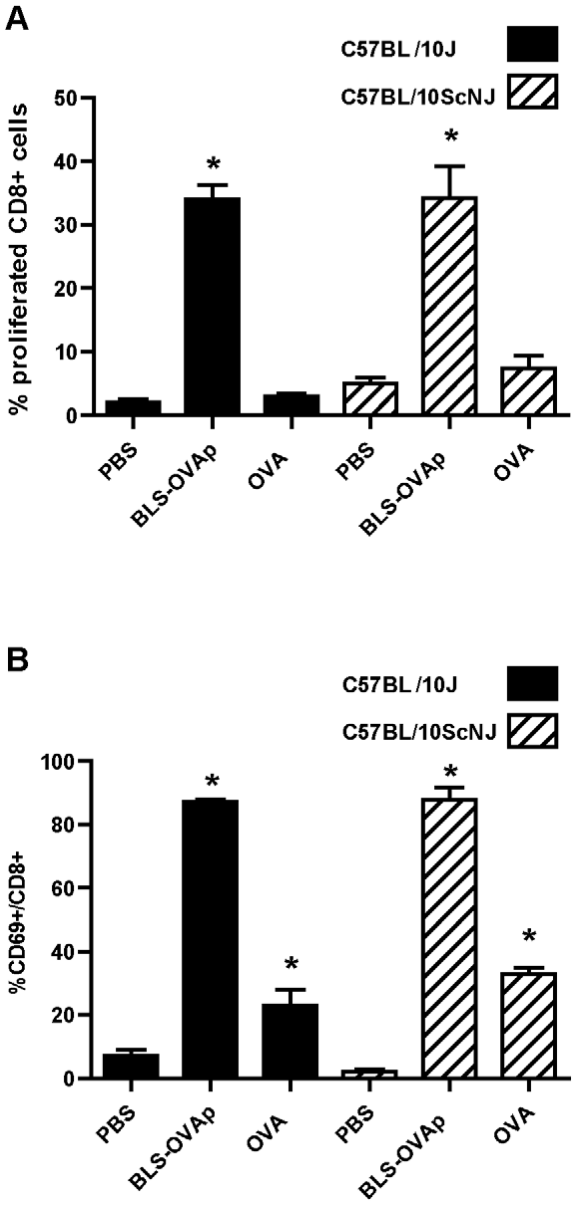
Cross presentation in TLR4-defficient mice. DCs from C57BL/10ScNJ (TLR4-defficient) or C57BL/10J (wild type) mice were incubated with BLS-OVA_257–264_ (BLS-OVAp), OVA or PBS. CD8+ cells from OT-I mice were stained with CFSE and added to the DCs. At 20 h, non-adherent cells were removed and analyzed by FACS. A: Bars represent the mean percentage of specific CD8+ cells that proliferated (gated on CFSE+cells)+SD (n = 6). B: Mean percentage of CD8+ cells expressing CD69+SD (n = 6). *p<0.05 compared to PBS. Data from two independent experiments have been pooled.

We have previously shown that BLS activates DCs *in vitro* and recruits DCs, B cells and CD8+ T cells *in vivo* via TLR4 [32]. We then evaluated if TLR4 was necessary for the induction of the proliferation and activation of specific CD8+ cells by BLS-OVA_257–264_. Due to differences in the genetic background of OT-I mice and TLR4-defficient mice [44]–[46], we could not perform the adoptive transfer assay. We assessed the ability of spleen-derived DCs from C57BL/10ScNJ (TLR4-defficient) or C57BL/10J (wild type) mice to cross-present BLS-OVA_257–264_ *in vitro* by following the response of naive OT-I lymphocytes.Purified DCs from spleen were incubated with BLS-OVA_257–264_ for 18 h and then fixed. Purified CD8+ cells from OT-I mice were stained with CFSE and added to the DCs. Twenty hours after incubation, the proliferation and the surface expression of CD69 of CD8+ cells were measured. DCs from TLR4-defficient mice induced similar levels of proliferation of OT-I cells than DCs from wild type mice (Fig. 4A).The percentage of CD8+ OT-I cells that expressed CD69+ was increased at the same level as when exposed to BLS-OVA_257–264_-stimulated wild type DCs, as shown by FACS analysis (Fig. 4B). Dendritic cells incubated with OVA did not induce the proliferation of OT-I cells. No significant differences were found when using DCs from C57BL/10J or C57BL/6J mice (not shown). We analyzed the expression of costimulatory molecules on the surface of the cultured DCs. As expected, BLS-OVA_257–264_ induced the activation of DCs from wild type mice and did not activate DCs from TLR4-defficient mice, as shown by CD40 and CD80 expression (not shown). In conclusion, these data show that the cross presentation of BLS-OVA_257–264_ is independent on the presence of a functional TLR4 on the DCs and on their activation state.

### BLS-OVA_257–264_ Induces a TLR4-Dependent Strong *In Vivo* Cytotoxicity

**Figure 5:**
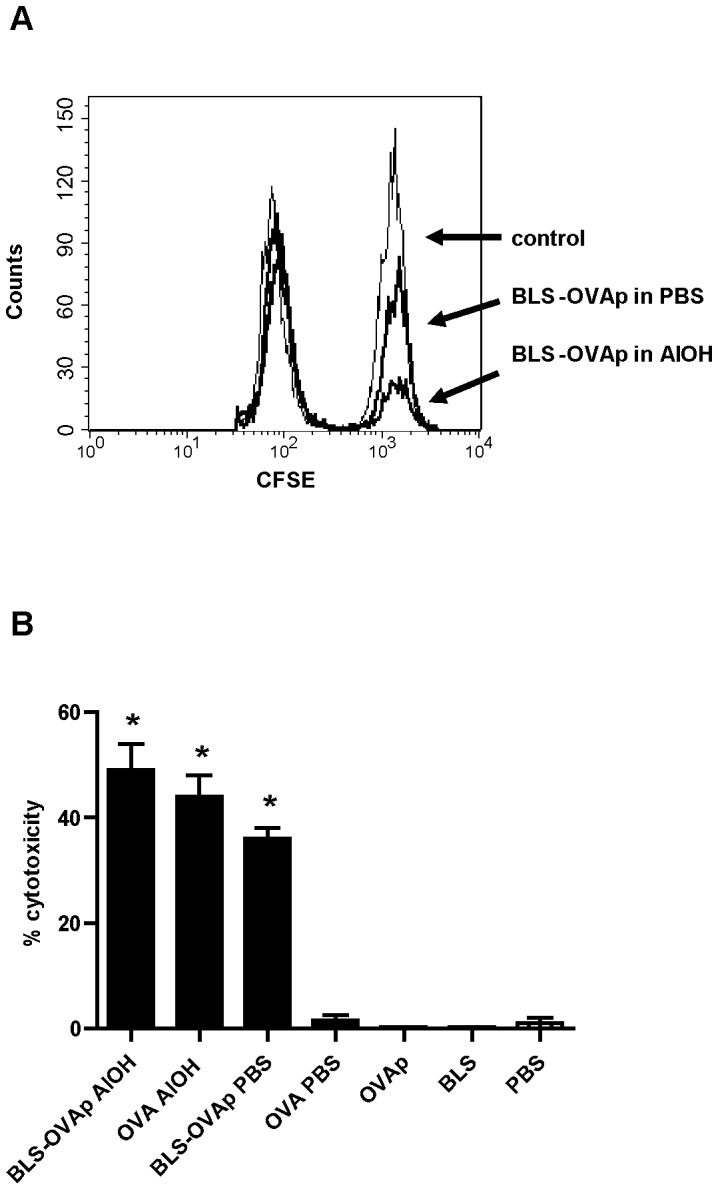
Specific cytotoxicity induced by BLS-OVA_257–264_. A: Representative overlayed histograms of CFSE^high^ and CFSE^low^ populations within CFSE+ cells from inguinal lymph nodes of C57BL/6J mice immunized with BLS-OVA_257–264_ (BLS-OVAp) in PBS, BLS-OVAp in AlOH or PBS (control). B: Bars represent the mean percentage+SD of specific cytotoxicity in lymph nodes of mice immunized with BLS-OVAp in AlOH, OVA in AlOH, BLS-OVAp in PBS, OVA in PBS, OVAp in PBS, BLS in PBS or with PBS (n = 12). *p<0.05 compared to control. Data from two independent experiments have been pooled (6 mice per group).

Finally, we performed an *in vivo* assay that allows the determination of antigen-specific cytotoxicity [37], [38]. To this end, C57BL/6J mice were immunized subcutaneously with BLS-OVA_257–264_. In parallel, naive C57BL/6J mice splenocytes were pulsed *in vitro* with peptide OVA_257–264_ and then incubated with a high concentration of CFSE (CFSE^high^). Unpulsed splenocytes were incubated with a lower concentration of CFSE (CFSE^low^). At day 6, immunized mice were intravenously inoculated with CFSE^high^- and CFSE^low^-labeled cells in equal amounts. After 18 h, the draining lymph nodes were removed, and FACS analysis was performed. Figure 5A shows representative overlayed histograms of CFSE fluorescence in cells from inguinal lymph nodes. Figure 5B shows the mean percentage of cytotoxicity calculated as described in materials and methods. Immunization with BLS-OVA_257–264_ in PBS induced a strong *in vivo* specific effector CTL response in C57BL/6J mice, as evidenced by the decrease in the number of CFSE^high^ cells (Fig. 5A). The number of CFSE^low^ cells remained invariable, demonstrating the specificity of the cytotoxic response. Immunization with the chimera with the adjuvants CFA, IFA (not shown) and AlOH also induced specific cytotoxicity, but only AlOH increased the percentage of cytotoxicity induced by BLS-OVA_257–264_ in PBS (Fig. 5A and B). Unlike BLS-OVA_257–264_, OVA did not induce cytotoxicity in the absence of adjuvants. As expected, immunization with BLS or OVA_257–264_ did not induce any cytotoxicity. In all groups, the results obtained for the paraaortic lymph nodes were similar to those obtained for inguinal lymph nodes (data not shown). These results clearly show that BLS-OVA_257–264_, even in the absence of adjuvants, specifically induces the cytotoxicity of OVA_257–264_-loaded cells.

**Figure 6:**
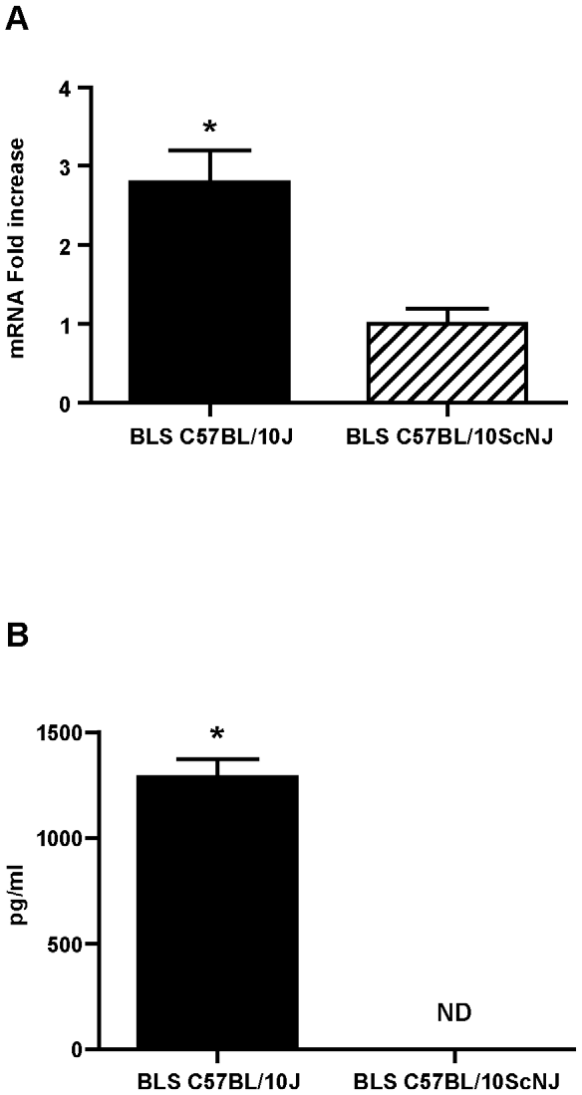
IFN-γ induced by BLS. A: C57BL/10J or C57BL/10ScNJ were immunized with BLS in the right hind footpad. At 48 h total RNA from draining lymph nodes was obtained. The expression of IFN-γ was determined by RT-PCR. Data are expressed as the fold increase of mRNA in draining versus non-draining lymph nodes (n = 6). B: C57BL/10J or C57BL/10ScNJ mice were immunized with BLS or PBS. Splenocytes were re-stimulated with BLS *in vitro*. IFN-γ in the supernatants were measured by ELISA. ND: Not detectable. Bars represent means+SDs (n = 6). *p<0.05 compared to control. Data of two independent experiments have been pooled.

**Figure 7:**
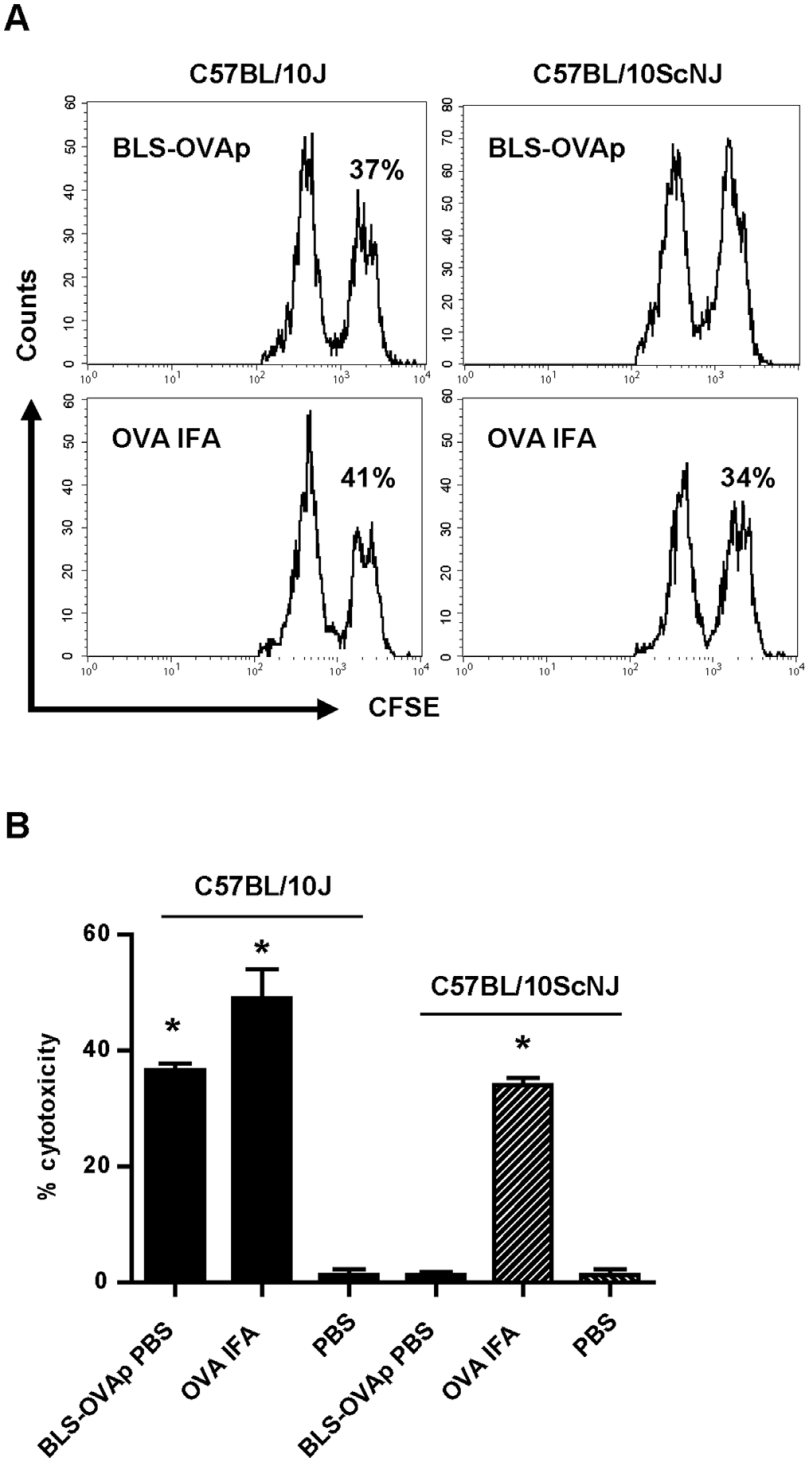
BLS-OVA_257–264_ induces a CTL response via TLR4. A: Representative histograms of CFSE^high^ and CFSE^low^ populations within CFSE+ cells from inguinal lymph nodes of C57BL/10J or C57BL/10ScNJ mice immunized with BLS-OVA_257–264_ (BLS-OVAp) or OVA in IFA. The percentage of specific lysis is shown. B: Bars represent the mean percentage+SD of specific cytotoxicity in lymph nodes of C57BL/10J or C57BL/10ScNJ mice immunized with BLS-OVAp in PBS, OVA in IFA or with PBS (n = 12). *p<0.05 compared to control. Data from two independent experiments have been pooled (6 mice per group).

It is well known that IFN-γ secretion is crucial for the generation of a cytotoxic response. Noteworthy, the carrier BLS by itself induces the production of IFN- γ via TLR4. Figure 6 show the levels of IFN-γ mRNA in the draining lymph nodes of wild type and TLR4-defficient mice immunized with BLS. The level of IFN-γ mRNA is increased in wild type mice but not in TLR4-defficient immunized mice (Fig. 6A). We also measured by ELISA the secretion of IFN-γ in splenocytes from immunized mice re-stimulated *in vitro* with BLS. As expected, only wild type splenocytes secreted IFN-γ when exposed to BLS (Fig. 6B). Thus, it is likely that this property of BLS would be extended to BLS-OVA_257–264_ and would contribute to induce high specific cytotoxic responses against the inserted peptide. To determine whether TLR4 signaling has a role in the induction of cytotoxicity, a CTL assay was performed in TLR4-defficient mice. To this end, C57BL/10ScNJ or C57BL/10J mice were immunized with BLS-OVA_257–264_. Splenocytes from wild type C57BL/10J mice, incubated with OVA_257–264_, were used as target cells. We observed that OVA_257–264_-specific cytotoxicity was completely abrogated in BLS-OVA_257–264_ immunized TLR4-defficient mice (Fig. 7). As a control, immunization with OVA in IFA induced cytotoxicity both in wild type and TLR4-defficient mice. These results show that the CTL response induced by BLS-OVA_257–264_ is dependent on TLR4.

## Discussion

To eliminate intracellular pathogens or to generate an anti-tumor response, vaccines require the induction of antigen-specific cytotoxicity. Successful generation of a cytotoxic response requires the presentation of peptides from internalized antigen by class I MHC molecules on APCs. A second signal composed of costimulatory molecules and cytokines, provided mostly by APCs, is required for T cell activation. Type I IFN and IL-12 serve as a third signal, facilitating CD8+ T cell proliferation, effector function and memory formation, [47] and it has been shown that CD27/OX40 can also serve as signal 3 mediators [48]. The absence of the second signal can result in the absence of a response or the immunological tolerance of specific T cells. Targeting exogenous antigens to immature DCs can induce tolerance; however, if the antigen is inoculated with an activation stimulus, the targeted DCs induce immunity [49], [50]. Pathogen-associated molecular pattern (PAMP) recognition through TLRs results in the activation of APCs and the production of a variety of pro-inflammatory mediators [51]. The outcome of antigen presentation by DCs depends on their activation status, such that TLR-induced DC activation produces a strong immune response, whereas steady-state antigen presentation leads to tolerance, thereby preventing the induction of an immune response against self antigens [52]–[54]. In this regard, Bachmann et al. [55], either packaging different TLR-ligands into virus-like particles or using mice deficient in two key molecules of TLR signaling, showed that an innate stimuli is necessary for the induction of a CTL response, but not for cross presentation. On the other hand, Oh et al. [56] showed that cross presentation of OVA is achieved only when the antigen is covalently attached to a TLR7 agonist, determining the success of protective CD8+ response. Koniaras et al. reported that OVA_257–264_ immunization of OT-I mice induced the proliferation and activation of specific CD8+ cells, but that these peptide-stimulated cells were not capable of inducing a cytotoxic response and eventually underwent apoptosis [57]. It has been postulated that the soluble peptide could bind the MHC I molecule directly on the cell surface, generating proliferation and a transient activation [58]. Nonetheless, in the absence of other signals, such as multivalent ligation of the TCR and co-stimulation, effector cells are not generated. It has been shown that injection of peptide OVA_257–264_ in IFA transiently activates CD8+ effector T cells, which eventually fail to undergo secondary expansion or to kill target cells as a result of a sustained and systemic presentation of the CTL peptides [59]. In this work, we showed that immunization of wild type mice with BLS-OVA_257–264_ in the absence of adjuvant induces the specific cytotoxicity of intravenously-transferred OVA_257–264_-loaded cells from wild type mice. Remarkably, BLS-OVA_257–264_ immunization induced the cytotoxicity of 36% of OVA_257–264_-loaded cells at 18 h of the transfer of the cells. We also showed that alum boosts the capacity of BLS-OVA_257–264_ to trigger T cytotoxic specific activity, may be implicating an increased activation of DCs. Specific cytotoxicity was not generated in mice immunized with the peptide alone, confirming that the phenotype of cells with effector functions was not generated unless the peptide was conjugated to BLS. The CTL assay in TLR4-defficient mice showed that BLS-OVA_257–264_ induces a specific CTL response via TLR4, as this response is completely abolished in the absence of a functional receptor in the immunized recipient mice. We showed that BLS induces the cross presentation of a peptide linked to its structure, stimulating a rapid activation and proliferation of specific CD8+ cells *in vivo*. BLS-OVA_257–264_ also increases the percentage of specific effector cells at 5 days of immunization. To study if TLR4 has a role in the cross presentation induced by BLS, we performed an *in vitro* assay. We showed that BLS-OVA_257–264_-stimulated DCs from TLR4-defficient mice induced the same levels of proliferation and activation of OT-I cells than BLS-OVA_257–264_-stimulated DCs from wild type mice. Thus, we can conclude that the ability of BLS to induce the cross presentation of peptide OVA_257–264_ does not rely on its capacity to signal through TLR4 and that the induction of cross presentation does not determine the generation of a cytotoxic response. Our results also show that an efficient cross presentation can occur despite the lack of activation of dendritic cells, as BLS-OVA_257–264_-stimulated TLR4-defficient DCs are not activated. Collectively, these and previously reported results show that the immunological response induced by BLS is in part regulated by TLR4 (DC activation and recruitment, cytokine production) but not at the level of antigen presentation. The secretion of IFN- γ and the activation of the DCs are presumably necessary steps for the induction of specific cytotoxicity.

The results presented here show that, unlike other subunit-based vaccines, BLS chimeras are extremely efficient in rapidly activating specific CD8+ lymphocytes and inducing significant cytotoxic activity. This efficiency would be based on the capacity of BLS to present antigens through the class I pathway and to deliver a second signal to dendritic cells through TLR4. The results presented here give a better understanding of the remarkable efficacy of BLS as a useful carrier in vaccine development. This knowledge will allow improvements in the development of acellular vaccines for clinical and veterinary use.
